# Physiological and cortical determinants of exercise tolerance in sedentary women with obesity

**DOI:** 10.3389/fspor.2026.1771921

**Published:** 2026-04-13

**Authors:** Asma Kacem, Abdullah Almheiri, Abdulqudos Ishaq, Foued Ftaiti

**Affiliations:** 1 Laboratory of Physiology, Faculty of Medicine Ibn El Jazzar, Sousse, Tunisia; 2 General Administration of Training, Sports Training Department, Dubai Police Academy, Dubai, United Arab Emirates

**Keywords:** women, obesity, exercise tolerance, fatigue, EEG, thermoregulation

## Abstract

**Background:**

Obesity significantly limits exercise tolerance, yet the specific contributions of central vs. peripheral fatigue remain debated. This study investigated whether early exhaustion in sedentary women with obesity is driven by a failure in central neural drive or by cumulative peripheral physiological strain.

**Methods:**

Twelve women with obesity (BMI 35.9 ± 4.0) and ten non-obese controls (BMI 22.3 ± 1.3) performed submaximal cycling at 60% of their maximal aerobic power until voluntary exhaustion. We simultaneously monitored cortical activity via EEG (alpha (α) and beta (β) wave power and the α/β ratio), cardiovascular response (HR), thermoregulation (Tty), and metabolic markers (lactate concentration and body mass loss).

**Results:**

Compared with non-obese participants, obese women experienced a significantly shorter exercise duration (∼36.8% lower). This was accompanied by a faster rate of fluid loss (1.2 ± 0.2 vs. 0.6 ± 0.2 kg), a significantly higher peak tympanic temperature (38.1 ± 0.3 vs. 37.7 ± 0.2 °C), and a lower peak heart rate at exhaustion (175.2 ± 5.3 vs. 186.2 ± 6.5 bpm). However, relative intensity reached at exhaustion was similar between groups (93.7 ± 2.9% vs. 96.1 ± 3.3% of HR max, respectively), indicating near-maximal cardiovascular strain in both populations. EEG analyses revealed no significant between-group differences in α activity, β activity, or the α/β ratio across exercise stages. In both groups, α activity decreased and the α/β ratio (index of central fatigue) also decreased from rest to exercise, reflecting increased cortical activation.

**Conclusion:**

These findings indicate that early fatigue in sedentary women with obesity is primarily a result of peripheral physiological overload specifically cardiovascular, thermal, and metabolic constraints rather than a lack of central cortical arousal. The similar relative physiological strain at exhaustion suggests that exercise tolerance is limited by reaching critical peripheral thresholds sooner than non-obese counterparts.

## 1 Introduction

Obesity, defined by excess body fat, represents a major global health crisis impacting all age groups (1–3). Excess fat mass inherently increases the energetic cost of movement and places significant, chronic strain on both the cardiovascular and metabolic systems. In youth, overweight and obesity are clearly linked to lower aerobic fitness, earlier fatigue, and greater cardiovascular stress during physical activity (4, 5). Similarly, in adults, impaired metabolic health is a key concern, with cardiorespiratory fitness serving as a robust long-term predictor of cardiometabolic risk (6).

Women with obesity commonly exhibit lower aerobic capacity, reduced muscle strength, and a heightened sensitivity to heat, factors which collectively severely limit exercise tolerance (7, 8). Sedentary obese women therefore face higher cardiovascular and thermal strain during exercise, making it difficult to sustain even moderate workloads (9, 10). Critically, excess adipose tissue acts as an effective thermal insulator, impeding heat dissipation and thereby accelerating fatigue.

Beyond mechanical and thermoregulatory constraints, obesity is known to disrupt hormonal and metabolic regulation. Disturbances in hormones like leptin, growth hormone, and related signaling pathways contribute to increased fatigue perception and reduced physical performance (11–13). Since these disturbances affect both muscle metabolism (peripheral) and brain function (central), fatigue in obesity likely arises from a complex interplay between the working muscle, the body's metabolic state, and central nervous system control.

While exercise is a vital tool for health (1, 14, 15), research often overlooks the brain's role in governing fatigue. Prolonged exertion is known to alter cortical activation; however, in populations with obesity, it remains unclear if exhaustion stems from a failure in central motor command or cumulative peripheral strain. In hot environments, an increase in the alpha-to-beta (alpha/beta) EEG ratio—an index of central fatigue is typically associated with shorter time to exhaustion (16). This increase was associated with shorter time to exhaustion and greater thermal and cardiovascular strain. Given that obesity acts as a thermal insulator, sedentary women may reach a peripheral “safety limit” before central drive is compromised (16, 17). Recent work emphasizes the crucial need to better distinguish these contributions during submaximal effort.

Obese women typically show lower cardiorespiratory fitness and reduced exercise capacity than lean women. Higher fat mass correlates with lower maximal oxygen uptake and impaired heart-rate responses during exercise (18), and reduced fitness is tied to greater inflammation and poorer metabolic health, especially in women (19). Furthermore, electrolyte balance and hematological status are important determinants of exercise tolerance. Disturbances in sodium, potassium, chloride, hemoglobin, and hematocrit induced by prolonged exercise and dehydration can impair endurance, oxygen delivery, and cognitive performance (20–22). These effects may be amplified by obesity and physical inactivity (16, 23).

Therefore, the aim of this study was to compare sedentary obese and non-obese women and investigate the mechanisms of fatigue during prolonged submaximal cycling under thermoneutral conditions. We hypothesized that early fatigue would result from combined peripheral and central mechanisms, with obesity accentuating the physiological and neural challenges associated with prolonged exercise to exhaustion.

## 2 Method

### 2.1 Participants

The study included twelve obese women (mean age 21.7 ± 2.2 years; body mass 95.4 ± 14.9 kg; height 162.9 ± 5.6 cm; BMI 35.9 ± 4.0 kg⋅m^−2^) and a control group of ten non-obese women (mean age 22.1 ± 2.0 years; body mass 60.3 ± 7.6 kg; height 164.4 ± 6.3 cm; BMI 22.3 ± 1.3 kg⋅m^−2^). Detailed anthropometric and functional characteristics of the participants, including body fat percentage, maximal aerobic power (MAP), and the 60% MAP workload, are summarized in Table 1.

**Table 1:** Anthropometric and physiological profiles of obese and non-obese women.

Parameter	Obese (*n* = 12)	Non-obese (*n* = 10)	*p*-value
A. Morphological profile			
Age (years)	21.7 ± 2.2	22.1 ± 2.0	NS
Height (cm)	162.9 ± 5.6	164.4 ± 6.3	NS
Weight (kg)	96.4 ± 12.1	60.3 ± 7.6	<0.001
BMI (kg·m^−2^)	35.9 ± 4.0	22.3 ± 1.3	<0.001
Body fat (%)	38.6 ± 3.4	19.5 ± 3.5	<0.001
B. Maximal aerobic capacity			
VO_2_max (mL·min^−1^·kg^−1^)	24.4 ± 6.8	32.8 ± 1.7	<0.01
HRmax (bpm)	187.0 ± 4	193.8 ± 3	<0.05
MAP (W)	214.9 ± 10.0	204.0 ± 22.4	NS
C. Experimental workload			
Submaximal Intensity (60% MAP, W)	128.7 ± 6.1	119.5 ± 15.7	NS

Values are presented as mean ± SD. BMI, body mass index; VO₂max, maximal oxygen uptake; MAP, maximal aerobic power; NS, not significant (*p* > 0.05).

All participants were classified as sedentary (IPAQ score < 150 min of moderate activity/week). To minimize any variance in baseline activity, we excluded anyone involved in structured sports or regular gym-based training. Participation was restricted to individuals who engaged in no more than 90 min of total moderate walking per week, ensuring that everyone fell strictly within a sedentary classification. We also standardized hydration by instructing participants to consume 500 mL of water the evening before and another 500 mL 2 h prior to their session. During the trials themselves, we permitted no fluid intake and used no external fan cooling; this allowed us to capture a natural physiological response. Finally, all participants wore standardized, lightweight athletic clothing to ensure the evaporative surface area was consistent across both groups.

All participants were fully informed of the nature and potential inconveniences associated with the experiment. Written informed consent was obtained from all participants prior to participation. Ethical approval was obtained from the appropriate research ethics committee (Committee for Human Protection in Biomedical Research of Sousse-Tunisia) and the study was performed in accordance with the ethical standards laid down in the 1964 Declaration of Helsinki. participants were not taking any medication or nutritional supplements. They were asked to refrain from physical activities for 24 h before every test and to avoid coffee and cigarettes on the testing day. All women were tested in the middle of the follicular phase (PF) of the menstrual cycle, specifically between the 4th and 8th days after menstruation.

### 2.2 Preliminary testing

One week before the cycling sessions, participants completed a preliminary trial to determine body composition and maximal aerobic power. Skin-fold thickness was measured at four sites: triceps, suprailiac, abdomen and thigh (24) using a calliper (Harpenden, France). The percentage of body fat was calculated according to Durnin and Womersley (25). Maximal aerobic power was determined using a progressive ergo-cycle protocol (Ergoline, Germany). Increasing load was performed according to a standardized and individualized protocol (26). In brief, after a 3-min warm-up at 20% of theoretical MAP (TMAP), power was increased by 8% of TMAP every minute until exhaustion. Cadence was maintained between 60 and 70 rpm. Exhaustion was defined as the inability to maintain cadence for >10 s despite verbal encouragement. VO_2_max was confirmed by a leveling-off in oxygen consumption, a respiratory exchange ratio—(RER) >1.1, and reaching peak HR within ±10 bpm of age-predicted maximum.

The maximal aerobic power was taken as the cycling power at which VO_2_max occurred. The participants’ ventilation (VE) and fractional concentrations of expired O_2_ and CO_2_ (FEO_2_ and FECO_2_) were measured continuously by a gas analyser (ZAN 600 Meβgreräte, Germany).

### 2.3 Experimental design

The exercise protocol was performed in a thermoneutral environment (N-Ex: ambient temperature of 22 °C ± 0.4 °C and air humidity of 53% ± 8%). It consisted of a submaximal cycling exercise performed at 60% of the participants’ individual MAP until a state of extreme physical fatigue causing the termination of exercise (exhaustion). The workload corresponding to 60% MAP was chosen specifically to equalize relative metabolic demand between participants. All testing took place in the morning (∼9:00 a.m.).

The cycling session began with a warm-up of 5 min at 40% of the determined MAP. This was followed immediately by cycling at 60% of MAP until exhaustion. The exercise duration was defined as the time limit at 60% of the MAP (*T*_lim_−60%). To ensure strictly controlled conditions, the submaximal sessions were conducted using the same electronically braked ergometer (Ergoline, Germany) utilized for the initial MAP determination. By operating the device in constant-power mode, the braking torque automatically adjusted to any fluctuations in pedaling cadence (RPM). This setup was essential to ensure the workload remained fixed at 60% of each participant's MAP, preventing a drop in resistance even as fatigue set in.

While VO_2_ and RPE were not recorded, we validated that this fixed mechanical load translated into a comparable internal strain through in-task monitoring of heart rate and terminal blood lactate. As shown in our results, both groups reached high-intensity physiological markers (exceeding 88% HRmax and 4.0 mmol·L^−1^ lactate), confirming that the targeted exercise intensity provided a significant and equivalent metabolic challenge for both the obese and non-obese participants.

During all sessions, participants benefited from continuous medical assistance, including monitoring of internal body temperature and blood pressure. Heart rate (HR), tympanic temperature (Tty), lactate concentration, body mass loss (BML), and electroencephalogram (EEG) were measured during the cycling exercise.

### 2.4 The electroencephalogram recording

The preparation, data acquisition, and analysis of the EEG signal followed procedures previously described (17, 27). The electrode was positioned over the prefrontal cortex at the F3 position (28). EEG was recorded for 90 s at rest, at the onset of the exercise, and immediately before exhaustion. Data were analysed using a “Δ Med” EEG analyser (Coherence 3 NT, France).

EEG signals were amplified and sampled at a frequency of 500 Hz. A power spectrum was calculated from the EEG signal using fast Fourier transformation. The areas of the power spectrum in the α-band (8–13 Hz) and β-band (13–30 Hz) were quantified as α and β activity, respectively. Subsequently, an α/β ratio was calculated. This ratio was used as an index of “fatigue” based on the rationale that a decrease in β activity and an increase in α activity would reflect decreased arousal (17). All α/β indexes were normalized to the value obtained during rest (α/β = 100%) before the exercise trial, eliminating trial-to-trial and person-to-person variation.

### 2.5 Determination of heart rate and tympanic temperature, and haematological parameters

Heart rate (HR) was monitored with a Polar NV monitor (Polar NV, Kempele, Finland), and body temperature was estimated via tympanic temperature (Tty) immediately before and after exercise. Previous studies have demonstrated that Tty provides a reliable measure of core temperature changes during exercise, evolving similarly to rectal temperature but detecting changes more rapidly (29, 30). The Tty was measured by a tympanic probe (T type thermocouple designed by INSERM, unit 103, Montpellier, France). This probe was carefully placed in the external auditory canal of the right ear until a slight pain indicated contact with the tympanic membrane (31).

Blood sampling was performed 3 min before and 3 min after each cycling exercise from the right cephalic vein while the participant was in a sitting position. Blood was collected in a simple dry tube and lactate concentrations were assessed using a whole-blood measuring device (L-Lactate Analyzer, Lactate Bayer, Sensor, France).

Body mass loss (BML) was determined from the pre- and post-exercise difference in mass between nude and dried body mass. Body mass was measured during all testing sessions using a force plate (Kistler 929 °C, Switzerland) with 1 g accuracy.

### 2.6 Statistical analysis

Descriptive statistics were calculated for all data and presented as means ± standard deviation (SD). α and β waves and the α/β ratio were analyzed at rest, at the onset of the exercise, and immediately before exhaustion using two-way ANOVA (time of EEG measurements, and group) with repeated measures. To compare the effect of “exercise” and “participant group” on endurance capacity, tympanic temperature, heart rate, BML, and lactate concentration, a two-way analysis of variance (ANOVA) with repeated measures was performed, taking into account the factors of pre- and post-exercise and participants’ corpulence classification (obese and non-obese). When significant main effects were found (*p* < 0.05), a *post-hoc* Scheffé multiple comparison test was applied to determine cell-to-cell differences. To address the risk of Type II errors and quantify the magnitude of observed differences regardless of *p*-values, we reported partial eta squared (*η*^2^*_p_*) for all main effects and interactions.

## 3 Results

### 3.1 Electroencephalogram (EEG) variations

Analysis of electroencephalogram (EEG) activity recorded at rest, at the onset of exercise, and at exhaustion revealed no significant between-group differences in the evolution of alpha (α) waves (*F* = 3.5; *p* = 0.08; *η*^2^*_p_* = 0.14), beta (β) waves (*F* = 0.7; *p* = 0.40; *η*^2^*_p_* = 0.03), or the α/β ratio (*F* = 2.9; *p* = 0.09; *η*^2^*_p_* = 0.11). While the *p*-value for the α/β ratio approached significance, the associated effect size indicates that group membership accounted for only a small-to-moderate portion of the variance in cortical arousal.

In both groups, alpha wave activity showed a significant decrease at the onset of exercise and at exhaustion compared with resting values (*F* = 78.6, *p* < 0.001; *η*^2^*_p_* = 0.80). This decrease signifies increased cortical arousal during exercise relative to rest. Beta wave activity demonstrated a significant decrease from exercise onset to exhaustion only in the non-obese group (*F* = 8.9, *p* < 0.001; *η*^2^*_p_* = 0.31). No significant changes in beta waves were observed across exercise stages in the obese group, and no significant differences were detected between onset and exhaustion when both groups were considered together.

The α/β ratio also decreased significantly at exercise onset and at exhaustion compared with rest in both groups (*F* = 42.1, *p* < 0.001; *η*^2^*_p_* = 0.68), reflecting a shift toward greater cortical activation/arousal during exercise. However, the magnitude of these decreases did not differ significantly between obese and non-obese participants (Table 2).

**Table 2:** Endurance performance and cortical EEG activity (alpha, beta and α/β ratio).

Parameter	Moment	Obese (*n* = 12)	Non-obese (*n* = 10)	*p*-value
Endurance time				
*T*_lim_ (min)	Total	30.1 ± 6.8	47.6 ± 8.4	<0.001
A. Absolute EEG (%)				
Alpha (α)	Rest	24.5 ± 6.4	30.7 ± 6.3	NS
	Onset	7.6 ± 3.3	4.2 ± 1.4	NS
	End	5.5 ± 1.6	10.9 ± 1.6	NS
Beta (β)	Rest	18.3 ± 4.1	15.6 ± 5.4	NS
	Onset	21.1 ± 2.8	25.1 ± 8.8	NS
	End	11.4 ± 5.4	14.7 ± 2.5	NS
α/β ratio	Rest	1.4 ± 0.5	2.2 ± 0.7	NS
	Onset	0.3 ± 0.1	0.2 ± 0.1	NS
	End	0.6 ± 0.3	0.8 ± 0.1	NS
B. Normalized EEG	(Rest = 100%)			
Alpha (α)	End	25.0 ± 8.7	37.9 ± 9.0	NS
Beta (β)	End	66.3 ± 33.6	105.4 ± 27.9	NS
α/β ratio	End	44.4 ± 22.8	40.9 ± 17.4	NS

Values are expressed as mean ± SD. *T*_lim_, time-to-limit (endurance time); α, alpha band; β, beta band; NS, not significant (*p* > 0.05). Part B displays the percentage of change from rest to the point of exhaustion.

### 3.2 Exercise duration (*T*_lim_−60%)

Time to exhaustion was significantly lower in obese compared with non-obese women (30.1 ± 6.7 vs. 47.6 ± 12.8 min; *F*_(1,20)_ = 17.1, *p* < 0.001; *η*^2^*_p_* = 0.46). Partial eta squared (*η*^2^*_p_*) was reported to quantify the magnitude of the between-group difference beyond statistical significance. This corresponds to a significant difference (*p* < 0.001) of approximately 36.8% lower endurance in the obese participants.

### 3.3 Tympanic temperature response

Mean tympanic temperatures recorded immediately before exercise were similar between groups (36.8 ± 0.2 °C for both). During the test, metabolic heat production and accumulation led to a significant rise in Tty in both groups (*F* = 238, *p* < 0.001; *η*^2^*_p_* = 0.54). At exhaustion, Tty reached 38.1 ± 0.3 °C (Delta Tty = +1.3 ± 0.3 °C) in obese participants and 37.7 ± 0.2 °C (Delta Tty =  +0.9 ± 0.2 °C) in non-obese participants (*p* < 0.001). The maximal Tty recorded at the end of the exercise showed a significantly higher increase (*p* < 0.001) in the obese compared to the non-obese group (*F* = 12; *p* < 0.001).

When calculating the rate of change over time, the obese group showed a significantly (*p* < 0.001) faster rise in temperature (0.043 ± 0.01 °C.min^−1^) vs. compared to the non-obese group (0.019 ± 0.005 °C.min^−1^).

### 3.4 Heart rate (HR) response

Resting HR was similar between groups (obese: 83.3 ± 6.3 bpm; non-obese: 85.7 ± 5.8 bpm). During submaximal cycling, HR increased significantly in both groups (*F* = 13.78, *p* < 0.001; *η*^2^*_p_* = 0.49). However, at the time of exhaustion (*T*_lim_), obese participants reached a significantly (*p* < 0.001) lower peak heart rate (175.2 ± 5.3 bpm) than non-obese participants (186.2 ± 6.5 bpm, Table 3).

**Table 3:** Cardiovascular and thermoregulatory responses during submaximal cycling.

Values are presented as mean ± SD. Parameter	Obese (*n* = 12)	Non-obese (*n* = 10)	*p*-value
Cardiovascular strain			
Resting HR (bpm)	83.3 ± 6.3	85.7 ± 5.8	NS
Peak HR (bpm)	175.2 ± 5.3	186.2 ± 6.5	<0.05
Intensity (% measured HRmax)	93.7 ± 2.9	96.1 ± 3.3	NS
HR kinetics (bpm·min^−1^)	3.05 ± 0.8	2.11 ± 0.6	<0.001
Thermoregulatory stress			
Resting Tty (°C)	36.8 ± 0.2	36.8 ± 0.2	NS
Peak Tty (°C)	38.1 ± 0.3	37.7 ± 0.2	<0.01
Rate of Tty Rise (°C·min^−1^)	0.043 ± 0.01	0.019 ± 0.01	<0.001
Hydration and fluid loss			
Absolute body mass loss (kg)	1.2 ± 0.2	0.6 ± 0.2	<0.001
Relative body mass loss (%)	1.25 ± 0.2	1.01 ± 0.3	<0. 05
Fluid loss velocity (kg·h^−1^)	∼2.4	∼0.75	<0.001

Values are presented as mean ± SD. HR, heart rate; Tty, tympanic temperature; HRmax, maximum heart rate achieved in preliminary ramp test; NS, not significant (*p* > 0.05).

The rate of heart rate increase per minute was significantly (*p* < 0.001) higher in the obese group (3.05 ± 0.8 bpm·min^−1^) than in the non-obese group (2.11 ± 0.6 bpm·min^−1^). When expressed as a percentage of the HRmax determined during the preliminary ramp test, both groups reached a comparable relative intensity at exhaustion (93.7 ± 2.9% vs. 96.1 ± 3.3%).

### 3.5 Body mass loss (BML)

Pre-exercise body mass was significantly higher in the obese group (*F* = 57.4, *p* < 0.001). While the absolute difference between pre- and post-exercise mass was relatively small within each group, the total BML was significantly (*F* = 147.7, *p* < 0.001, *η*^2^*_p_* = 0.88) higher in obese participants (1.2 ± 0.2 kg) compared to the non-obese group (0.6 ± 0.2 kg). This corresponded to a significantly greater relative dehydration of 1.25 ± 0.2% in the obese group vs. 1.01 ± 0.3% in the control group (*p* < 0.05). Notably, the rate of fluid loss was substantially higher in the obese group (∼2.4 kg.h^−1^) compared to the control group (∼0.75 kg.^h−1^).

### 3.6 Blood lactate concentration

At rest, lactate concentrations were comparable between obese (1.8 ± 0.4 mmol⋅L^−1^) and non-obese participants (2.0 ± 0.5 mmol⋅L^−1^). Following exercise, the maximal blood lactate concentration was significantly lower in the obese group (3.9 ± 0.3 mmol⋅L^−1^) compared with the non-obese group (5.1 ± 0.2 Mmol⋅L^−1^) (*F* = 110.3, *p* < 0.01; *η*^2^*_p_* ≈ 0.85).

## 4 Discussion

This study investigated why sedentary women with obesity often experience early fatigue. We compared their peripheral physiological responses with central cortical activity during prolonged, submaximal cycling. Our findings revealed a significantly lower exercise tolerance in the obese group. While the termination of exercise was voluntary, the terminal physiological markers specifically the near-maximal heart rates and elevated blood lactate levels confirm that participants reached a state of true physiological failure. The significant thermal and cardiovascular strain suggests that “exhaustion” was not merely a perceptual threshold, but a protective response to a body nearing its homeostatic limits. The lower absolute heart rate at exhaustion in the obese group does not indicate a lack of effort, but rather an accelerated approach to their physiological limit. The significantly steeper HR kinetics, combined with the fact that they reached over 93% of their measured HRmax in nearly 40% less time, suggests that cardiovascular strain was a primary driver of earlier exhaustion.

This was closely linked to higher thermoregulatory strain, greater dehydration, and altered cardiovascular and metabolic profiles. Interestingly, EEG data showed that cortical activation remained comparable between both groups, both at rest and during exercise. These findings suggest that early fatigue in sedentary obese women arises predominantly from peripheral physiological overload rather than a lack of central neural drive. While we cannot statistically rule out subtle differences in central drive due to our sample size, the robust and significant differences in peripheral markers (Tty, BML, and HR) suggest that these factors reached critical thresholds well before any statistically detectable divergence in cortical arousal occurred.

### 4.1 Peripheral physiological constraints and exercise intolerance

Obese participants achieved a significantly shorter exercise duration and showed greater body mass loss, a larger increase in tympanic temperature, lower peak heart rate, and reduced maximal blood lactate accumulation. These results align with established literature showing that excess fat mass increases the mechanical and metabolic demands of exercise altering the body's ability to dissipate heat, thus accelerating fatigue (7–10, 32).

The pronounced body mass loss in women with obesity points toward accelerated dehydration, a critical factor in exercise-induced fatigue. In our study, the obese group experienced a significantly higher rate and magnitude of fluid loss compared to the non-obese group. This rapid fluid loss is well-documented to reduce plasma volume, which in turn compromises cardiovascular function and limits oxygen delivery to the working muscles (20–22), effects that are significantly amplified when combined with thermal stress (33, 34).

This accelerated loss reflects the high sweat rate required to counter the rapid rise in core temperature seen in the obese participants. While absolute mass loss can be influenced by baseline weight, the significantly higher relative loss (1.25%) and the vastly higher velocity of loss (∼2.4 vs. ∼0.75 kg·h^−1^) suggest a more acute physiological challenge. This rapid fluid shift out of the intravascular space-occurring in roughly 30 min for the obese group likely triggered a critical cardiovascular and thermoregulatory strain much sooner than in the control group.

Furthermore, the lower peak blood lactate concentrations recorded at exhaustion in obese participants suggest that they stopped exercising earlier, implying altered metabolic regulation due to obesity-related hormonal and mitochondrial disturbances (11–13). Comparable alterations in lactate kinetics and substrate utilization have previously been reported in obese women (35).

Moreover, in sedentary obese individuals, the anaerobic threshold or maximal lactate steady state is typically reached at a lower relative percentage of maximal aerobic power, indicating that exercise performed at 60% MAP may already represent a supra-threshold intensity.

### 4.2 Cardiovascular and thermal constraints

Heart rate increased significantly (*p* < 0.001) in both groups. However, the significantly (*p* < 0.001) lower peak heart rate at exhaustion in the obese group reflects an earlier attainment of limiting physiological strain.

Attenuated heart-rate responses are a known issue in obese women with low cardiorespiratory fitness, correlating directly with reduced exercise tolerance (18, 19). This blunted chronotropic response, possibly combined with reduced stroke volume adaptation (36, 37), becomes a major limiting factor when paired with increased thermal and hydration stress, driving earlier voluntary exhaustion.

This accelerated approach to physiological limits is further evidenced by our thermal data. The obese group exhibited a faster and greater rise in tympanic temperature, likely driven by the fact that adipose tissue acts as a thermal insulator that limits convective heat loss. When combined with a fluid loss nearly double that of the control group (1.2 vs. 0.6 kg), the resulting thermal strain and hemoconcentration appear to induce voluntary exhaustion before the central nervous system reaches a state of “shut down.”

We recognize that the higher peak tympanic temperature in the obese group may reflect a combination of both higher absolute metabolic heat production and impaired heat dissipation. Since the obese group worked at a slightly higher absolute intensity (128.7 ± 6.1 W) compared to the controls (119.5 ± 15.7 W), their total heat production was likely greater. However, the rate of temperature rise in the obese group was more than double that of the non-obese group (0.043 vs. 0.019 °C·min^−1^). This disproportionate rise, coupled with nearly double the fluid loss (1.2 vs. 0.6 kg), suggests that even if production was higher, the body's inability to effectively dissipate that heat compounded by the insulating effect of adipose tissue was the critical factor limiting their exercise tolerance.

### 4.3 Central responses and EEG activity

**Figure 1:**
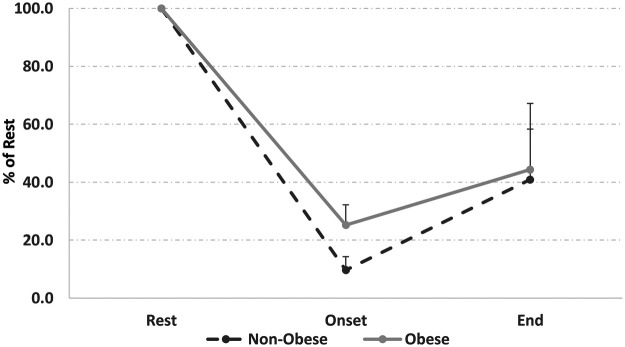
α/β ratio in obese and non-obese participants at rest, at exercise onset, and at exhaustion during submaximal cycling exercise. Values are normalized to resting values (100%).

The EEG analyses showed no significant differences in α activity, β activity, or the α/β ratio between groups. In both obese and non-obese participants, α activity decreased from rest to exercise and the α/β ratio also declined during exercise (Figure 1). This common pattern reflects sustained cortical activation in response to the physical challenge, indicating that the central nervous system maintained its arousal and motor drive (17, 20, 27). Accordingly, central cortical arousal, as measured at F3, was not the primary differentiating factor between groups. This finding aligns with preserved central neural engagement during submaximal exercise under thermoneutral conditions (38, 39).

Although the overall EEG patterns were similar, a subtle difference was noted: β activity decreased toward exhaustion only in the non-obese group. This might suggest a dynamic adjustment in cortical strategy for the non-obese group as they neared failure, potentially related to increased sensory input or cognitive effort. However, this difference is minor when considering the robust overall finding: despite the obese women stopping ∼37% earlier due to severe peripheral strain, their EEG indices of central drive were not disproportionately altered compared to the non-obese women. This strongly implies that a failure of central neural activation was not the primary mechanism for early fatigue in this population.

These findings support the conclusion that early fatigue in sedentary obese women arises predominantly from peripheral physiological overload rather than a primary impairment in central neural regulation.

The combined impact of increased mechanical load, impaired heat regulation, dehydration, a blunted cardiovascular response, and reduced metabolic flexibility provides a comprehensive explanation for their diminished performance.

This interpretation aligns with that emphasize the role of strong peripheral afferent feedback overwhelming the central cortical arousal, rather than isolated central failure (40, 41). While central mechanisms certainly modulate how hard the effort feels, they do not appear to be the primary rate-limiting factor during prolonged submaximal cycling in this population.

## 5 Limitations

Although the sample size is relatively modest, it is consistent with previous studies utilizing complex, multi-dimensional protocols involving continuous EEG and thermal monitoring. However, future studies with larger cohorts are warranted to confirm these patterns across different obesity phenotypes.

Regarding the intensity of the workload during the cycling exercise, it should be recognized that the 60% MAP workload utilized in this study represents a “hard load” that borders on the Maximal Lactate Steady State (MLSS) or Lactate Threshold 2 (LT2). This is clearly evidenced by our end-test markers, which showed that both groups reached more than approximately 88% of their HRmax, with blood lactate concentrations (BLC) reaching ∼4.1 mmol⋅L^−1^ for the obese group and ∼5.2 mmol⋅L^−1^ for the controls. These values are highly consistent with the onset of blood lactate accumulation (OBLA), an intensity where a physiological steady state is difficult to sustain and the body becomes increasingly susceptible to cardiovascular drift and heat storage.

We also acknowledge that using a single exercise-to-exhaustion trial without a prior familiarization session can introduce intra-individual variability. While constant-load tests are sensitive to daily fluctuations, the sheer scale of the difference found (36.8%), supported by a strong effect size (*η*^2^*_p_* = 0.46), points toward a robust physiological reality. To mitigate this, we took extensive care to stabilize the testing environment, including specific controls for hydration, time of day (09:00 a.m.), and the participants’ menstrual phase.

Regarding data collection, heart rate and tympanic temperature were not recorded at continuous 5-minute intervals. This choice was made to minimize external interference with the participants’ focus and to protect the integrity of the EEG signal. However, we recognize that the absence of these increments prevents a more granular analysis of the physiological kinetics during the transition from heavy to severe exercise.

Finally, we acknowledge that our study was not specifically powered for equivalence testing of EEG parameters. The non-significant findings in cortical activity should therefore be interpreted with caution; the lack of a *p*-value below 0.05 does not definitively prove that central drive is identical between groups. However, the marked disparity in effect sizes where peripheral strain indicators showed nearly total variance coverage (e.g., *η*^2^*_p_* = 0.88 for BML) compared to the much lower values for EEG variables (*η*^2^*_p_* = 0.11 for α/β) supports our primary conclusion: that peripheral constraints are the more dominant and statistically reliable drivers of earlier exhaustion in this population.

Future research should include larger, more diverse populations, including male participants and different age groups, to determine if these thermal and cardiovascular responses are universal across the obesity spectrum. In addition, direct biomarkers such as plasma osmolality or urine specific gravity should be incorporated to more precisely quantify the degree of intracellular and extracellular dehydration.

## 6 Conclusion

Our results suggest that early fatigue in sedentary women with obesity is due to peripheral physiological overload rather than a failure of cerebral motor signals or a lack of motivation.

While prefrontal cortical arousal remained comparable between groups, suggesting that motivational drive was maintained, the significantly earlier exhaustion in the obese group appears to be driven by an accelerated attainment of peripheral physiological limits.

This physiological strain resulted from accelerated thermoregulatory and fluid loss kinetics, combined with an elevated cardiovascular response that limited the heart's pumping capacity relative to the metabolic demand.

## Data Availability

The raw data supporting the conclusions of this article will be made available by the authors, without undue reservation.
